# Metabolic Reprogramming in Metastatic Melanoma with Acquired Resistance to Targeted Therapies: Integrative Metabolomic and Proteomic Analysis

**DOI:** 10.3390/cancers12051323

**Published:** 2020-05-22

**Authors:** Laura Soumoy, Corentin Schepkens, Mohammad Krayem, Ahmad Najem, Vanessa Tagliatti, Ghanem E. Ghanem, Sven Saussez, Jean-Marie Colet, Fabrice Journe

**Affiliations:** 1 Laboratory of Human Anatomy & Experimental Oncology, Faculty of Medicine and Pharmacy, University of Mons, 7000 Mons, Belgium; laura.soumoy@umons.ac.be (L.S.); sven.saussez@umons.ac.be (S.S.); 2 Laboratory of Human Biology & Toxicology, Faculty of Medicine and Pharmacy, University of Mons, 7000 Mons, Belgium; corentin.schepkens@umons.ac.be (C.S.); vanessa.tagliatti@umons.ac.be (V.T.); jean-marie.colet@umons.ac.be (J.-M.C.); 3 Laboratory of Oncology and Experimental Surgery, Institut Jules Bordet (ULB), 1000 Brussels, Belgium; mohammad.krayem@bordet.be (M.K.); ahmad.najem@bordet.be (A.N.); gghanem@ulb.ac.be (G.E.G.)

**Keywords:** metastatic melanoma, targeted therapies, resistance to drugs, metabonomics, proteomics, metabolic switch, cancer metabolic reprogramming

## Abstract

Treatments of metastatic melanoma underwent an impressive development over the past few years, with the emergence of small molecule inhibitors targeting mutated proteins, such as BRAF, NRAS, or cKIT. However, since a significant proportion of patients acquire resistance to these therapies, new strategies are currently being considered to overcome this issue. For this purpose, melanoma cell lines with mutant BRAF, NRAS, or cKIT and with acquired resistances to BRAF, MEK, or cKIT inhibitors, respectively, were investigated using both ^1^H-NMR-based metabonomic and protein microarrays. The ^1^H-NMR profiles highlighted a similar go and return pattern in the metabolism of the BRAF, NRAS, and cKIT mutated cell lines. Indeed, melanoma cells exposed to mutation-specific inhibitors underwent metabolic disruptions following acute exposure but partially recovered their basal metabolism in long-term exposure, most likely acquiring resistance skills. The protein microarrays inquired about the potential cellular mechanisms used by the resistant cells to escape drug treatment, by showing decreased levels of proteins linked to the drug efficacy, especially in the downstream part of the MAPK signaling pathway. Integrating metabonomic and proteomic findings revealed some metabolic pathways (i.e., glutaminolysis, choline metabolism, glutathione production, glycolysis, oxidative phosphorylation) and key proteins (i.e., EPHA2, DUSP4, and HIF-1A) as potential targets to discard drug resistance.

## 1. Introduction

Melanoma is the deadliest form of skin cancer [1]. Its incidence has been steadily increasing for the last 20 years and it represents the first form of cancer among people aged 25 to 29 [2]. This type of cancer is particularly difficult to treat, especially when diagnosed at advanced stages. Indeed, the 5-year survival for metastatic melanoma is less than 15% [3]. Most melanoma cells are radio and chemoresistant, mainly due to their melanin production. The current treatments for metastatic melanoma rely on targeted therapies and, more recently, immunotherapies. The most used targeted therapies act on the MAPK pathway, which is mutated in NRAS and BRAF in about 25% and 60% of melanoma patients, respectively [4]. Nevertheless, the major challenge with such inhibitors is that melanoma cells possess an hypermutable genome and activate many alternative signaling pathways, leading to acquired resistances to such therapies [5]. Indeed, the great majority of patients with metastatic melanoma treated with a targeted therapy develop resistance in weeks or months following the onset of treatment [6]. Strengthened by this fact, the concept of combinatorial therapy has imposed itself.

Altered metabolism is a hallmark of cancer [7]. This can be partially explained by the fact that some protooncogenes, such as KRAS or c-myc, act on key metabolic enzymes [8,9,10,11]. The metabolic patterns significantly differ in cancer cells compared to healthy ones. Notably, the energy substrates and building blocks often differ between cancer and healthy cells. This adaptation is mandatory to support a higher demand in energy and biosynthetic precursors needed by malignant cells in order to sustain their malignant progression, high rates of proliferation, and invasion [12]. The major metabolic pathways modified in cancer cells are glycolysis [13,14], oxidative phosphorylation [15], and glutaminolysis [16]. This metabolic reprogramming also affects the level of oxidative stress in cancer cells and can impair their antioxidant capacities [17]. For a decade, many researchers studied the metabolic reprogramming occurring in cancer cells in order to better understand and characterize these processes, with a special focus on the identification of potential biomarkers and new therapeutic targets [18,19]. In this respect, metabolomic approaches are believed to play a major role in the management of cancer patients by helping the development of more personalized therapies [20].

To date, studies on melanoma cell metabolism under targeted therapies or after resistance acquisition mainly focused on cells treated with BRAF inhibitors. These studies indicated that BRAF inhibitors used in BRAF-mutated melanoma cells led to a decreased expression of glycolytic enzymes associated with lower glucose consumption. These studies indicated that the resistance to BRAF inhibitors was linked to an increased oxidative metabolism associated with an increased mitochondrial dependency [21,22,23]. They also demonstrated a higher mitochondrial biogenesis in resistant cells. These metabolic changes developed along with a switch from glucose to glutamine as the main source for energy synthesis. An overexpression of glutamine transporters was also observed in resistant cells [21,22,23]. Regarding these results, BPTES, a glutaminolysis inhibitor, was used on melanoma cells either sensitive or resistant to BRAF inhibitors. The results indicated that resistant cells were more sensitive to glutaminolysis inhibition than sensitive ones [21,23]. Another important finding from the literature is the observation that resistant cells produce more glutathione and overexpress many antioxidative genes, indicating stronger antioxidant defenses in these cells, in order to survive in a more oxidative environment due to increased oxidative metabolism [17]. 

MAPK and PI3K-AKT, the two major oncogenic signaling pathways in melanoma, have been extensively studied. It turns out that resistance to BRAF and MEK inhibitors mainly occurs through new modifications in these signaling pathways [24,25,26,27,28]. Acting on cell signalization alone still fails to overcome the acquisition of such resistance. More recent studies have demonstrated many interconnections between metabolic fluxes and the RAS-MAPK and PI3K-AKT-mTOR pathways [29,30], highlighting the importance of an integrative approach of metabolism and cell signalization.

In the present work, we evaluated which metabolic pathways are affected in melanoma cells that are exposed to targeted therapies, in cells with acquired resistance to these drugs and their parental counterpart. The aim was to understand the metabolic changes occurring among melanoma lines, either sensitive or resistant to targeted therapy, with the perspective of identifying new targets for combinatorial studies in order to overcome or at least delay the development of acquired resistance. As the current literature concerning metabolic changes in resistant melanoma cells mainly focuses on BRAF mutated cells, we decided to expand our studies to other important mutational profiles. We used three different cell lines with frequent gene mutations in melanoma. The two mutually exclusive BRAF and NRAS mutations account for about 85% of the mutations affecting the MAPK pathway [31,32]. The cKIT mutation, only occurring in 2%–3% of all melanoma cases, is mutated or amplified in about 30% of mucosal melanoma [5,33].

## 2. Results

### 2.1. ^1^H-NMR Cellular Extract Signature of the Three Types of Cell Lines with Acquired Resistance

#### 2.1.1. Identification of the Discriminant Metabolites Using Multivariate Data Analysis

We performed our analyses on three different cell lines: HBL (^D820Y^cKIT), MM074 (^V600E^BRAF), and MM161 (^Q61R^NRAS). ^1^H-NMR spectra of the cellular extracts of each cell line were generated. For each cell line, three main experimental conditions were conducted. The first one consisted of sensitive cells free of any drugs (MM074, MM161, HBL). The second one corresponded to sensitive cells exposed to their corresponding targeted therapy for a short time (24 and/or 72 h) for results purposes (MM074 DABRA 24 h, MM074 DABRA 72 h, MM161 PIMA 72 h, HBL DASA 72 h). The latter included the resistant counterpart for each cell line considered. Those resistant cells were produced in-lab using a continuous exposure of melanoma cells to increasing concentrations of their corresponding targeted therapy for at least 12 weeks (MM074-R DABRA, MM161-R PIMA, HBL-R DASA).

The ^1^H-NMR spectra of cellular extracts (Figures S1–S3) were binned into small subregions of 0.04 ppm stepwise (called descriptors), the AUC of each subregion was integrated, and all numerical data exported to an Excel table. Then, each signal was independently normalized, dividing each descriptor by the total spectral area under the curve (AUC) signal of their corresponding cell line and experimental group.

**Figure 1:**
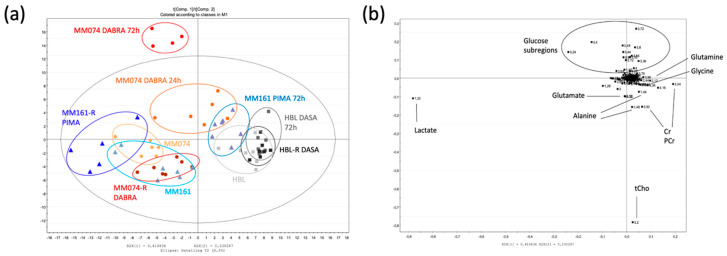

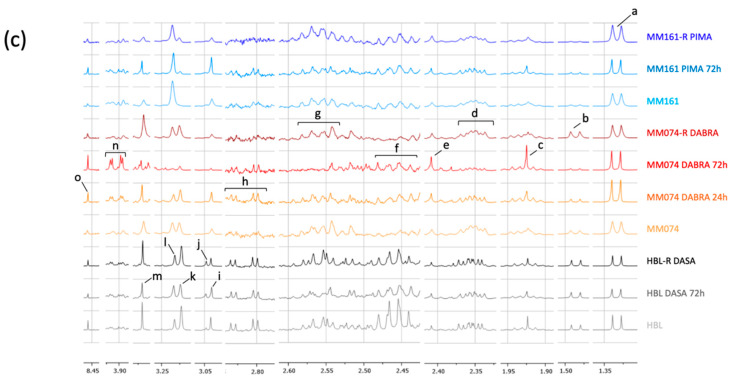
Scores plot (**a**) and loadings plot (**b**) of the partial least square analysis (PLS-DA) of the drug-free (MM074, MM161, HBL), short-time drug-exposed (MM074 DABRA 24 h, MM074 DABRA 72 h, MM161 PIMA 72 h, HBL DASA 72 h) and long-time drug-exposed (MM074-R DABRA, MM161-R PIMA, HBL-R DASA) cellular extracts (R^2^X = 0.929; R^2^Y = 0.527; Q^2^cum = 0.415). (**c**) NMR spectra of the IC compartments highlighting the discriminant-identified metabolites. For the sake of completeness, spectra were normalized against the total AUC to visualize the relative intensities of each metabolite between the groups. The metabolites were identified and tagged as follows: a: Lactate; b: Alanine; c: Acetate; d: Glutamate; e: Succinate; f: Glutamine; g: Glutathione (GSH); h: Aspartate; i: Creatine (Cr); j: Phosphocreatine (PCr); k: Phosphocholine (PCho); l: Glycerophosphocholine (GPC); m: Glycine; n: Glucose; o: Formate.

A supervised partial least square analysis (PLS-DA) was next applied to the data to highlight the most reliable discriminating metabolites in group separation, resulting in a scores plot (Figure 1a) and its matching loadings plot (Figure 1b). Focusing on the scores plot, the first principal component t [1] discriminated the HBL groups from the others, with, however, a notable exception. Indeed, the ‘MM161 PIMA 72 h’ group found itself distributed in the HBL zone, highlighting the Pimasertib effect on the MM161 metabolic signature. The second principal component t [2] clearly separated both the ‘MM074 DABRA 24 h’ and ‘MM074 DABRA 72 h’ groups from the others, underlying the Dabrafenib effect on the MM074 metabolic response. The matching loadings plot (Figure 1b) displays the six most discriminating metabolites (including metabolites, carbohydrates) with a variable importance in the projection (VIP) value above 1, namely lactate, glycine, glutamine, glutamate, glucose, and alanine, as well as two groups of metabolites, creatine and phosphocreatine (Cr/PCr), and the total choline content (tCho) (Figure 1c).

#### 2.1.2. Additional Spectral Investigations

**Figure 2:**
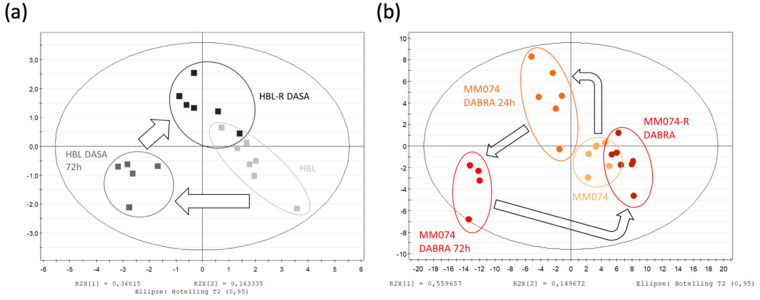

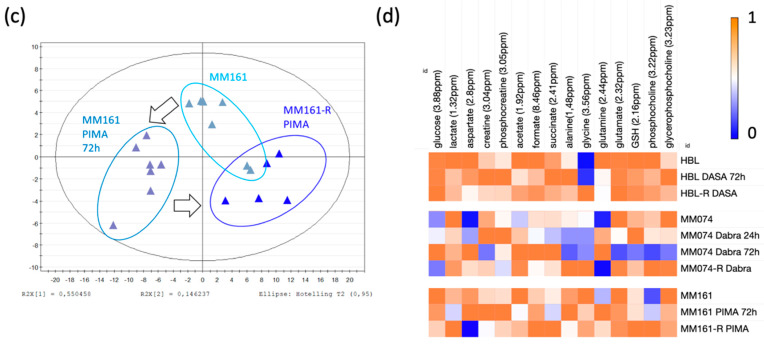
Scores plots highlighting the evolution of the IC metabolic signatures from an initial parental to a resistant status, in the case of mutant cKIT (**a**), mutant BRAF (**b**), and mutant NRAS (**c**) acquired resistance. Arrows were used to draw the metabolic switches followed by sensitive cells during resistance acquisition and exposed or not to targeted treatments. (**d**) Heatmap of the 15 IC discriminant metabolites identified between the 3 considered cell lines. Data normalization from [0 to 1] for each metabolite, enabling the metabolic signature investigation within and between the groups.

^1^H-NMR intracellular (IC) spectra were further investigated using the MestRenova software. Indeed, some metabolites can be hidden due to spectral overlapping or low intensity. Using the MestRenova Peak Picking tool, numerical data were successfully collected and separated from the two metabolic groups identified in the loadings plot. Thus, the resonances of PCho and GPC, as well as Cr and PCr, were successfully separated from each other. Furthermore, five extra metabolites with a low intensity were detected thanks to additional spectral investigations, being acetate, aspartate, glutathione (GSH), succinate, and formate. The global signature was next processed through statistical analyses (Tables S1–S3) and displayed into a heatmap (Figure 2d).

#### 2.1.3. Independent Evaluation of the NRAS, cKIT, and BRAF Resistance Signatures

The scores plot can be used as a tool to visualize and evaluate metabolic changes. Indeed, the localization of each sample on the plot depends on the metabolic composition of the medium. In this context, the trajectory of different group samples on the plot reflect metabolic changes between groups. To fully view the metabolic resistance trajectory occurring in each resistance case, independent multivariate data analysis models were generated and discussed below. At first sight, a similar two-step pattern was detected in each case. First, a metabolic shift occurred during acute drug exposure as compared to the initial unexposed condition. More interestingly, a longer exposure allowed partial recovery of the basal metabolic profile, as can be seen by the relocation of resistant groups close to the unexposed ones. This “roundtrip” pattern is marked by arrows on the scores plot.

Focusing on the HBL scores plot (Figure 2a), the previously described pattern is perfectly highlighted. Some explanations of the HBL metabolic signature shift due to the acute Dasatinib exposure can be found in the heatmap (Figure 2d). Thus, when comparing the acute signature to the unexposed one, increases in creatine, phosphocreatine, succinate, alanine, and GPC levels, as well as decreases in lactate, glutamine, formate, acetate, and phosphocholine levels were recorded. Then, when the HBL resistant signature is compared to the two others, it gives clues on the metabolic recovery process. Thus, most of the metabolites returned to their unexposed levels, but lactate, aspartate, and glutamine remained as affected as in the acute exposure condition.

Considering the BRAF-mutated cell line MM074, exposed signatures were collected at 24 and 72 h, enabling the short-term kinetic evaluation of the Dabrafenib exposure (Figure 2b). A progressive increase in the levels of glucose, aspartate, acetate, formate, and glutamine, as well as a progressive decrease in the levels of alanine, glycine, glutamate, GSH, PCho, and GPC were noticed. The creatine shuttle (Cr/PCr) appeared to be disrupted only at 72 h. In the resistant signature, most of the metabolites returned to their basal unexposed levels. Interestingly, both aspartate and acetate conserved the same levels from 72 h (acute study) up to longer term (chronic study) exposure to Dabrafenib.

Finally, the “roundtrip” metabolic pattern of the NRAS-mutated cell line MM161 was screened (Figure 2c). When comparing the acutely exposed cell signatures to the unexposed one, decreases in the levels of lactate, acetate, and succinate, and increases in the levels of creatine, phosphocreatine, and glutamine were noticed. The resistant signature showed a return to basal unexposed levels for most of the metabolites, except for the glutamine levels, which were sustained to the levels reached during acute exposure. Furthermore, exclusive metabolic changes were detected in the resistance signature, with increased levels in GSH, PCho, and succinate, associated with a drop in aspartate levels.

### 2.2. ^1^H-NMR Extracellular Signature Investigations

**Figure 3:**
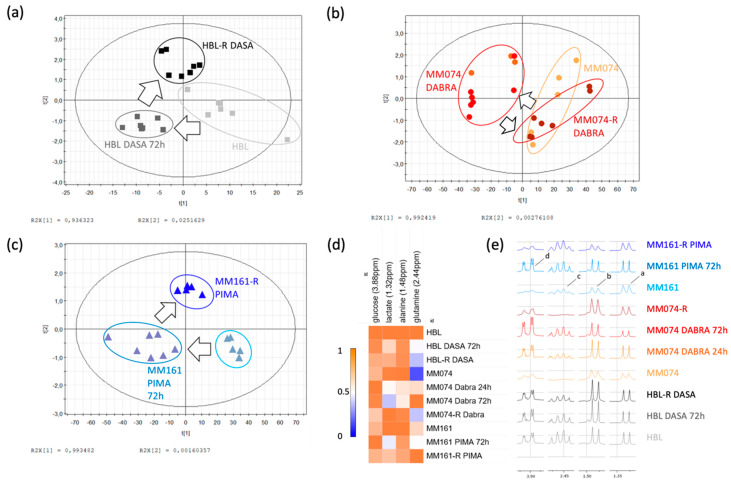
Scores plots showing the evolution of the culture media composition from an initial to a resistant status, in the case of cKIT (**a**), BRAF-V600E (**b**), and N-RAS (**c**) acquired resistance. Arrows were used to draw the metabolic path followed by sensitive cells during resistance acquisition. (**d**) Heatmap of the 4 EC discriminant metabolites identified between the 3 considered cell lines and experimental conditions. (**e**) NMR spectra of the EC compartments highlighting the discriminant identified metabolites, tagged as follows: a: lactate; b: alanine; c: glutamine; d: glucose.

Culture media were also analyzed using the combination of ^1^H-NMR spectroscopy and multivariate data analysis. As it turned out, the independent PLS-DA analysis of the extracellular (EC) returned the same “roundtrip” pattern as the one already described in the IC signature (Figure 3a–c). Four metabolites were responsible for the group separation, being lactate, alanine, glutamine, and glucose, as shown in the heatmap (Figure 3d) and spectra (Figure 3e).

One of the most significant changes observed in the HBL culture medium composition is the decrease in the glutamine concentration during resistance acquisition, most likely due to higher cellular intake. These resistant cells also slightly reduced their glucose use and lactate production.

Considering the MM074 and MM161 EC compartments, the medium composition was modified in the acute exposed condition, where a decrease in both the glucose and glutamine concentrations as well as a decrease in the lactate concentration were recorded. Alanine levels were also exclusively reduced in the MM074 culture medium in the Dabrafenib acute exposure condition.

### 2.3. Protein Microarray and Interactome Analyses

For protein microarray analysis, 447 antibodies were probed with the three cell lines of this study under two experimental conditions, lines with acquired resistance (MM074-R, MM161-R, HBL-R) and their parental drug-sensitive counterparts (MM074, MM161, HBL). We used the log2 transformed data (Table S4), enabling the fold-change (FC) analysis of this panel of protein-relative intensities between the sensitive and resistance conditions.

**Figure 4:**
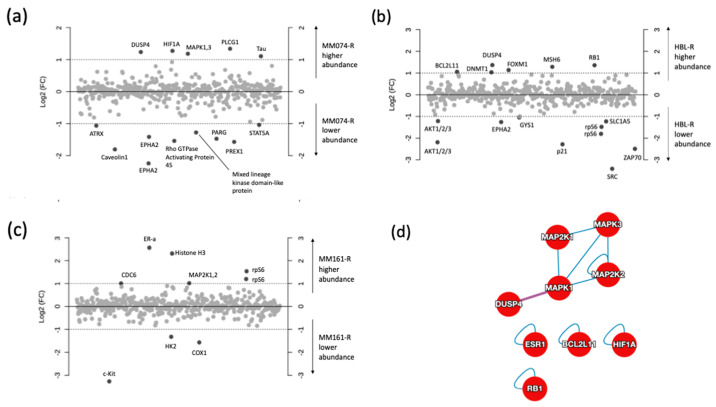

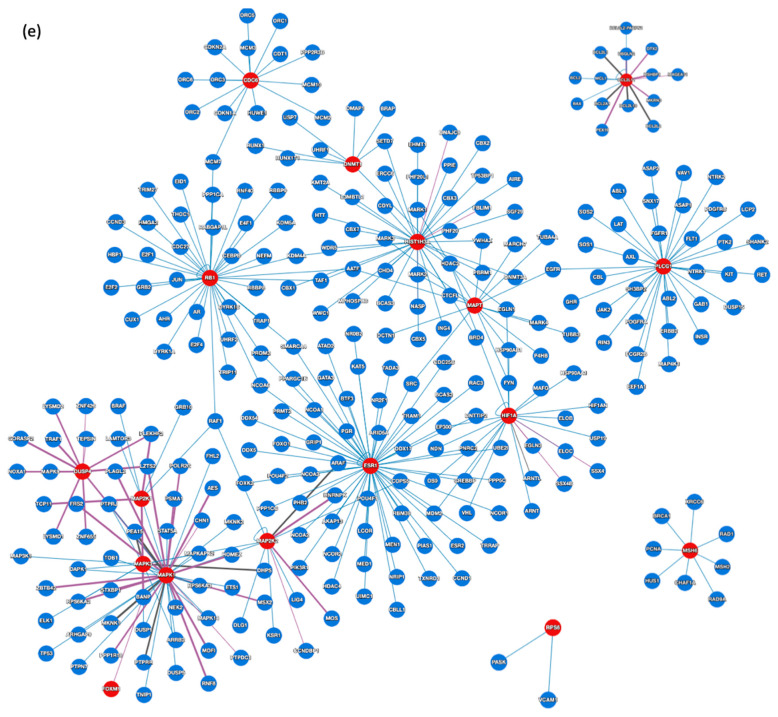
Log2 fold-change plots comparing relative protein levels between the resistant and sensitive counterpart of the MM074 (**a**), HBL (**b**), and MM161 (**c**) cells lines. Protein–protein interactome (PPI) of the highest abundant proteins identified the three resistant cases, highlighting the interactions shared between the 18 imputed proteins (**d**), or expanded to known interactors (**e**).

The first plot (Figure 4a) shows the comparison of the MM074-sensitive and -resistant conditions, with the identification of 13 proteins with a log2 (FC) above 1. The analysis successfully indicated that MM074-R presented higher abundances in DUSP4, HIF-1α, MAPK, PLCG1, and Tau (MAPT) proteins, as well as reduced amounts of ATRX, Caveolin 1 (CAV1), EPHA2, Rho GTPase activating protein 45 (ARHGAP45), mixed lineage kinase domain-like protein (MLKL), PARG, PREX1, and STAT5A.

Considering the HBL-resistant cell condition (Figure 4b), higher amounts of BCL2L11, DNMT1, DUSP4, FOXM1, MSH6, and RB1, and decreased levels of Akt1/2/3, EPHA2, GYS1, p21 (CDKN1), rpS6, SRC, and ZAP-70 were recorded.

Finally, a bench of nine proteins with a modified abundance were highlighted in the MM161-resistant case thanks to the fold change analysis (Figure 4c). Thus, increased levels of CDC-6, ER-α (ESR1), histone H3, MAP2K1/2, and rpS6 and decreased levels of c-kit (KIT), HK2, and CDK1 were observed.

Using the Human Reference Protein Interactome Mapping Project, protein–protein interaction (PPI) analysis was next applied on the 18 most abundant proteins identified in the resistant cases to get some understanding of the resistance mechanisms. The first PPI network (Figure 4d) returned the interactions between the imputed targets, with 9 of the 18 involved targets. The next PPI network (Figure 4e) clustered the abundant proteins in the resistant counterparts with their interactors, and generated a total of 306 proteins with 331 interactions and an average node degree of 2.15.

### 2.4. Integration of Metabonomics and Protein Microarray Data

**Figure 5:**
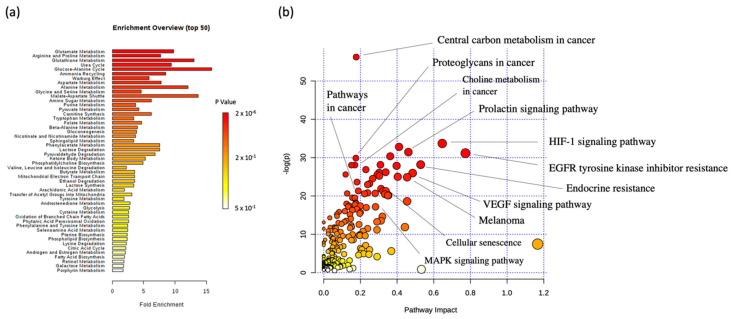
(**a**) Connection of the 15 significantly changed metabolites to metabolic pathways using MSEA. (**b**) Joint pathway analysis of the significantly changed metabolites and proteins, with highlighted cancer-related pathways highlighted. The size and color of the circular symbols highlight the pathway impact and *p*-value, respectively.

All 15 discriminant metabolites were selected for a metabolic set enrichment analysis (MSEA) using the MetaboAnalyst 4.0 online software. MSEA can connect the metabolites set presenting relative concentration variations to biological pathway(s), thus improving data interpretation. The resulting bar chart (Figure 5a) highlighted the potential biological pathways related to the metabolic data set. Pathways were classified depending on the number of hit(s), corresponding to the number of the 15 imputed metabolites recovered from currently available referenced pathways. Depending on the number of imputed metabolites retrieved from a whole pathway, *p*-values were calculated and used for classification. Focusing on the pathways that come out from the MSEA analysis, from a cancer point of view, special attention is paid to: ‘Glutamate metabolism’, ‘glutathione metabolism’, ‘Warburg effect’, ‘aspartate metabolism’, ‘alanine metabolism’, ‘malate-aspartate shuttle’, ‘alanine metabolism’, and ‘glycine and serine metabolism’.

A joint pathway analysis was also performed using both discriminant metabolic and protein levels. The analysis returned a metabolome view (Figure 5b), where relevant pathways to the melanoma cancer topic were highlighted.

## 3. Discussion

The aim of this work was to combine both metabonomic and proteomic approaches in order to better understand the resistance adaptations occurring in metastatic melanoma exposed to targeted therapies and to identify new targets paving the way for novel combinatorial drug strategies. We used three different cell lines with frequent gene mutations in melanoma (BRAF, NRAS, or cKIT).

The metabonomic analysis of HBL cells indicates that the treatment of these HBL cells with Dasatinib leads to a disruption of their metabolism in first place, rapidly followed by a metabolic adaptation to survive under Dasatinib chronic exposure. The same dynamic evolution of metabolism is observed in both IC and EC compartments. To understand how these cells can survive under chronic treatment and become resistant, we were particularly puzzled by the behaviors of IC lactate, aspartate, and glutamine, as well as EC lactate and glutamine. Both lactate and glutamine (IC and EC) levels decreased after a short exposure to Dasatinib and stabilized after resistance acquisition. The aspartate level decreased slightly after acute exposure but more intensively after chronic treatment. Lactate is the end-product of glycolysis and is overproduced in many cancer cells under the Warburg effect [34]. Its decreased production could be linked to an inhibition of glycolysis by Dasatinib, as already reported in chronic lymphocytic leukemia [35]. As lactate levels stay low in lines with acquired resistance, in both IC and EC compartments, we suspected resistant cells to be less dependent on glycolysis for their energetic needs. This reprogramming could lead to an increased glutaminolysis as suggested by the lower glutamine levels in both acute and chronic exposures to Dasatinib. Glutamine is used as another important energetic fuel in many cancer cells [36], and cases of glutamine addiction have already been described in melanoma with resistance to BRAF inhibitors [21,23]. Finally, the decreased aspartate level can be related to a decreased use of the malate-aspartate shuttle for mitochondrial ATP production [37], or an increased anaplerotic use of aspartate [38]. Regarding the joint pathway analysis, the ‘EGFR tyrosine kinase inhibitor resistance’ strongly indicates adaptive mechanisms in those resistant cells. Indeed, resistant cells express less AKT, SRC, EPHA2, or rpS6. This led us to the hypothesis that resistant cells rely more on the downstream effectors of the MAPK and PI3K/AKT pathways in order to keep their oncogenic properties. This could favor an escape from some regulators acting on upper proteins of these pathways, such as NF1, COT1, or PTEN.

The metabolomic analysis of the BRAF mutated cell line MM074 exposed to Dabrafenib (1µM) for 24 and 72h indicated a metabolic switch in the IC score plot, followed by a return to a previous state when becoming resistant. The same “roundtrip” pattern was also observed in the EC compartment. Once again, we focused on those metabolites that did not follow the “roundtrip” evolution after resistance development, namely aspartate and alanine. They both increased after short exposure to 1µM Dabrafenib and kept relatively high levels after resistance acquisition. As already mentioned, an increase in the aspartate level can be indicative of a higher production of mitochondrial ATP [39]. It has long been known that BRAF mutation in melanoma cells confers them high glycolytic properties [39,40]. Studies have also demonstrated that BRAF inhibitors act on glycolysis by downregulating many glycolytic enzymes [17,23]. The same effect can be seen in our results, with increased levels of EC glucose and decreased levels of EC lactate after exposure to Dabrafenib. The adaptation to chronic Dabrafenib exposure led to an increase in mitochondrial ATP production that has already been documented in melanoma [22]. Another study also indicated that aspartate can be a growth-limiting metabolite in tumors [41]. Regarding alanine levels, a decrease under short exposure to drug was also observed followed by a very significant raise along with resistance development. An alanine increase can be correlated to an increase of protein synthesis [42]. Regarding the joint pathway analysis, reinforcement of the MAPK and HIF-1α signaling is strongly suspected in the MM074-R, a phenomenon already well documented in the literature [43,44,45]. Similar to the results in HBL-R cells, EPHA2 expression was also decreased in MM074-R cells, indicating a loss of MAPK regulation.

Finally, metabolomic analysis of the NRAS mutated cell line MM161 under either short or chronic exposure to the MEK inhibitor (1 µM Pimasertib) indicates the same switch and return pattern. In this case, the metabolites of interest are mainly found in the IC with an increase in the glutamine level after a short exposure that lasted after resistance development, pointing to a higher activity of glutaminase enzymes. Indeed, a major role of glutaminases as oncogenic drivers has been highlighted in many cancer types [46,47,48]. Their implication in drug resistance mechanisms has already been demonstrated in leukemia and glioma [49,50]. The increased conversion rate of glutamine to glutamate is a marker of increased glutaminolysis in these cells under treatment and after resistance development. We made the hypothesis that while acquiring resistance to the MEK inhibitor, these cells would switch from glycolysis to glutaminolysis as their main source of energy, but glutamine consumption could also be linked to increased antioxidant defenses. Indeed, glutamate does not reach its untreated level after resistance development, but this could be explained by its use for GSH synthesis. Indeed, GSH levels are strongly increased in MM161-R cells. Similar increased antioxidant capacities after resistance development to MEK inhibitors have been reported in BRAF mutated melanoma cells [17]. We made the hypothesis that while acquiring resistance to the MEK inhibitor, these cells would switch from glycolysis to glutaminolysis as their major source of energy. This is supported by the decreased expression of hexokinase II (HKII) observed in our RPPA analysis. Slightly contradictory findings between the reduced expression of HKII and the IC lactate level (indicating a glycolysis still active in MM161-R cells) could be explained by the strong effect of Pimasertib on glycolysis, although during the short exposure. Indeed, lactate production is strongly decreased after 72 h of Pimasertib exposure. Several studies indicated an apoptosis induction in many cancer types after HKII inhibition [49,51,52]. We made the assumption that MM161 cells could decrease their HKII levels in order to adapt and be able to escape the Pimasertib effect on its glycolytic mechanism. Similar to the results reported in HBL-R cells, aspartate levels dropped in MM161-R cells. High phosphocholine levels can be linked to a higher activity of the choline kinase, which catalyzes the transformation of choline into phosphocholine, which was highlighted by our joint pathway analysis and described in many types of metastatic tumors [53,54,55]. Interestingly, a study indicated that the overexpression and activation of choline cycle enzymes are mediated by oncogenic signaling pathways, such as the RAS-MAPK and PI3K-AKT [56]. This is in agreement with what we already know about the changes in the MAPK and PI3K-AKT pathways after resistance acquisition in melanoma cells [3]. This is of particular interest because this study also indicated the involvement of choline metabolism enzymes in the regulation of oncogenic signaling pathways [56]. In addition to its metabolic role in the TCA cycle, succinate can act as a communication signal molecule via its membrane receptor GPR91 whose activation can lead to increased PI3K-AKT and MAPK signaling pathways [57,58]. This is in line with our RPPA analysis that indicates an overexpression of the different MAP kinases in the MM161-R cell line, as well as the decrease of the upstream effector cKIT.

Based on a better understanding of the mechanisms of resistance, the aim of our work was to find new biomarkers linked to drug resistance development and/or new targets, allowing such resistance to be delayed or overcome. We studied three melanoma cell lines with representative mutations in order to find a common pattern of resistance to different targeted therapies. Regarding the metabolism, the common point between our different cell lines is that while acquiring resistance, cells tend to switch to a metabolism relying more on glutamine and mitochondria for ATP production. This opens some interesting perspectives to disrupt this resistant metabolome by using mainly two kinds of metabolic inhibitors. First, glutaminolysis [59] could be targeted by glutaminase inhibitors, such as the small inhibitor CB-839, that already showed promising results in resistant myeloma [60], esophageal carcinoma [61], and ovarian cancer [62]. Another possibility could be the use of agents targeting mitochondrial ATP, which has also already showed promising results in melanoma cells with acquired resistance to BRAF inhibitors [63] and should be tested in melanoma cell lines with acquired resistance to MEK or cKIT inhibitors. Mutation-related specific changes also need to be investigated. For example, the increase of the choline metabolism in MM161-R cells could be targeted by choline kinase inhibitors that can also negatively moderate the MAPK signaling pathway [56]. Regarding protein expression, the most interesting targetable protein is EPHA2 whose expression is decreased in both HBL-R and MM074-R cells. As EPHA2 is a modulator of the MAPK pathway, its early inhibition may delay the development of drug resistance. Such inhibitors have already shown an antitumoral effect in vitro and in vivo in colorectal and lung cancers [64,65].

## 4. Materials and Methods

### 4.1. Cell Lines and Culture

Metastatic melanoma cell lines were derived from tumors by the Laboratory of Oncology and Experimental Surgery at Institut J. Bordet, Université libre de Bruxelles (Brussels, Belgium). For the work, we selected 3 cell lines harboring different mutations: HBL (^D820Y^cKIT), MM074 (^V600E^BRAF), and MM161 (^Q61R^NRAS). These lines are sensitive to targeted therapies, but we also worked with their resistant counterparts. To develop these resistances, cells have been chronically exposed to increasing doses of targeted therapies for 12 weeks (0.01–1 µM) by M. Krayem and A. Najem. This model mimics what happened in patients developing resistances to targeted therapies. Acute drug exposure effects were generated by exposing sensitive cells to 1 µM of their corresponding drug during 72 h. Due to Dabrafenib’s impact on the MM074-sensitive cell density, a shortened time-point of 24 h of exposure was also considered.

Cells were grown in Ham-F10 medium (Lonza, Bâle, Switzerland) supplemented with 10% fetal bovine serum and 1% penicillin/streptomycin (both from Life Technologie, Carlsbad, CA, USA) at 37 °C in a humidified 95% air and 5% CO_2_ atmosphere. The cultures were determined to be free of Mycoplasma contamination using PCR-based detection.

### 4.2. Effectors

The BRAF inhibitor Dabrafenib, used for BRAF mutated cells, and the MEK inhibitor Pimasertib, used for NRAS mutated cells, were from Selleck Chemicals (Houston, TX, USA). The tyrosine kinase inhibitor Dasatinib, used for cKIT mutated cells, was from Bristol-Myers (New York, NY, USA).

### 4.3. ^1^H-NMR Spectroscopy

Before sample collection, 675-mm^3^ flasks of each cell line were cultivated to confluency. Concerning the culture media collection, they were replaced 48h before sample collection. They were then collected and stored at −20 °C until analysis. Concerning the cell collection, we washed them twice with cold DPBS (4 °C) (Life technologies, Carlsbad, CA, USA) and used 3 mL of cold methanol to quench their metabolism. Finally, cells were collected using a scraper. Prior to −80 °C storage, cell pellet was de novo quenched using a liquid nitrogen immersion for a few seconds.

Before ^1^H-NMR samples’ preparation, an additional methanol:water:chloroform 1:0.9:1 extraction was required for cell pellet preparation, to separate the polar metabolites from macromolecules. The resulting polar phase containing the metabolites held a mix of methanol and water, which interacted with the NMR signal. The solvents were therefore dried using a SpeedVacuum.

For ^1^H-NMR sample preparation, either the cellular extracts or culture media were mixed with phosphate buffer (0.2 M Na_2_HPO_4_/0.04M NaH_2_PO_4_, pH 7.4), previously prepared in a mixture of H_2_O/D_2_O (80:20). For the intracellular samples, 700 µL of buffer were added to the dried cell pellets, whereas 250 µL of buffer were mixed with 500 µL of the culture media. To remove residual macromolecules, samples were centrifuged for 10 min at 13,000 g. Next, 650 µL of each supernatant were mixed with 50 µL of a solution of Trimethylsilylpropanoic acid (TSP) solubilized in D_2_O, 7mM for cell pellets, and 14 mM for culture media. 

For ^1^H-NMR spectra acquisition, the previous described samples were loaded into 5-mm NMR tubes. Spectra acquisition was processed on a Bruker 600-MHz Advance spectrometer, using the NOESYPRESAT-1D pulse sequence and 256 scans. 

Both the phases and baseline of the obtained spectra were corrected using the MestreLab Research 10.0.2 software (Mestrelab Research, S.L, Santiago de Compostela, Spain). Next, spectra were merged into one page using the Stack tool, and the water peak region ranging from 4.20 to 5.20 ppm excluded. After ensuring good spectra alignment, spectra were binned into small subregions of 0.04 ppm width, and the area under the curve (AUC) of each descriptor calculated. Each 0.04-ppm width AUC was normalized to the total spectrum AUC.

Binned spectra were investigated using the SIMCA-P+ 12.0 software (Umetrics, Umeå, Sweden). To ensure the quality of the spectra processing, principal component analysis (PCA) was always carried out on the dataset prior to partial least square discriminant analysis (PLS-DA). The quality of the PLS-DA models was ensured by the two parameters R^2^ and Q^2^ values, based on the Umetrics recommendations. The supervised PLS-DA technique enabled variable importance in projection (VIP) calculation, with ones greater than the 1 value retained for further analyses.

The lack of sensitivity of the multivariate data analysis, as well as metabolites overlapping within the 0.04 ppm width, can hide some valuable information. Therefore, a semi-quantification analysis of the spectra within the MestRenova software was essential and returned additional spectral areas of interest. Next, the selected spectral areas were identified as metabolites using the Chenomx NMR suite software (version 8.1.1) and the human metabolome database (HMDB) [66].

Identified metabolites were then submitted to statistical analysis. First, the precise AUC of each metabolite was calculated within MestRenova using the Peak Picking tool. Then, the obtained numerical data were submitted to a Kruskal–Wallis test followed by a Dunn test. The considered *p*-values were Holm-corrected. 

The relative intensity of each discriminant metabolite was illustrated as a heatmap using the Morpheus online software.

### 4.4. Protein Microarray

Protein microarray was performed by RPPA analysis at the Functional Proteomics Core Facility at The University of Texas MD Anderson Cancer Center [67].

### 4.5. Protein–Protein Interactions (PPI)

Protein–protein interaction studies were conducted according to the Human Reference Protein Interactome Mapping Project [68].

### 4.6. MSEA and JOINT Pathway Analysis

Connection of the discriminant metabolites to metabolic pathways was performed using the Metabolic Set Enrichment Analysis (MSEA) tool from the online available software Metaboanalyst 4.0 [69], using the Homo Sapiens Pathway Library as a reference.

Data integration of both metabolite and protein sets was performed using the Joint Pathway Analysis of MetaboAnalyst, with the Homo Sapiens Pathway Library.

## 5. Conclusions

As metastatic melanoma is still one of the deadliest forms of cancer with a 5-year survival below 15% and as there is a lack of therapies effective in the long term due to the hypermutable genome of melanoma cells inducing the development of resistance to targeted therapies, it is crucial to better understand how these mechanisms take place. It is now clear that metabolic reprogramming plays a key role in resistance development. Our study indicated that common patterns took place in cells with different mutations exposed to different molecules of targeted therapy. As we showed that these common patterns indicate a particular role of glutaminolysis, glycolysis, and oxidative phosphorylation, we successfully targeted these processes and showed the disruption of the metabolome associated with the resistance to various targeted drugs.

Our integrative metabolomic and proteomic approach also pointed out the importance of cross-talk between metabolic enzymes and oncogenic drivers in the MAPK and PI3K-AKT pathways. This once again highlights the importance of integrative studies involving metabolic approaches in order to better understand the resistance mechanisms occurring in cancer cells.

## Supplementary Materials

The following are available online at https://www.mdpi.com/2072-6694/12/5/1323/s1, Figure S1. Typical cellular extracts 1H-NMR spectra of the MM074 cell line, Figure S2: Typical cellular extracts 1H-NMR spectra of the MM161 cell line, Figure S3: Typical cellular extracts 1H-NMR spectra of the HBL cell line, Table S1: Intracellular (A) and extracellular (B) metabonomic signatures of the MM161 cells in various experimental conditions, Table S2: Intracellular (A) and extracellular (B) metabonomics signatures of the MM074 cells in various experimental conditions, Table S3: Intracellular (A) and extracellular (B) metabonomics signatures of the HBL cells in various experimental conditions, Table S4: Median centered Log2 data of the relative protein levels between the HBL, MM074 and MM161 resistant and sensitive counterparts.
