# Supplementary material for: Metabolic Reprogramming in Metastatic Melanoma with Acquired Resistance to Targeted Therapies: Integrative Metabolomic and Proteomic Analysis
Source: Cancers (Basel). 2020 May 22;12(5):1323. doi: 10.3390/cancers12051323 (PMC7280989; doi:10.3390/cancers12051323)
Supplement: Supplementary file 1 [file cancers-12-01323-s001.pdf]

Supplemental Materials

Metabolic Reprogramming in Metastatic Melanoma with Acquired Resistance to Targeted Therapies: Integrative Metabolomic and Proteomic Analysis

Laura Soumoy, Corentin Schepkens, Mohammad Krayem, Ahmad Najem, Vanessa Tagliatti, Ghanem E. Ghanem, Sven Saussez, Jean-Marie Colet and Fabrice Journe

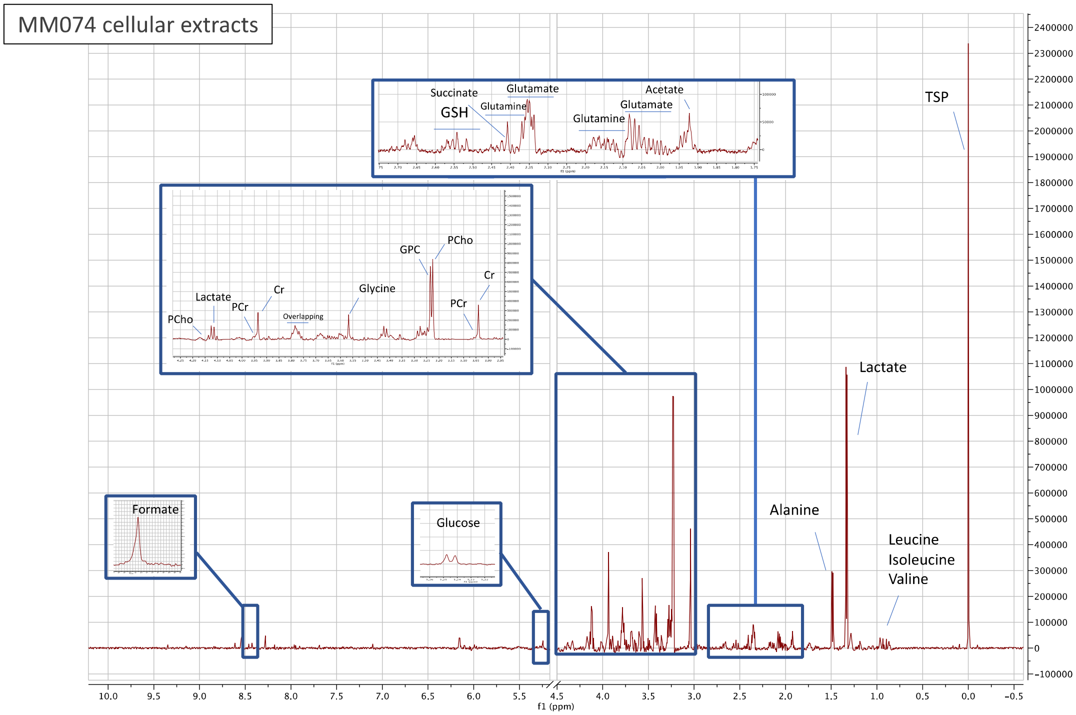

Figure S1. Typical cellular extracts ^{1}H-NMR spectra of the MM074 cell line.

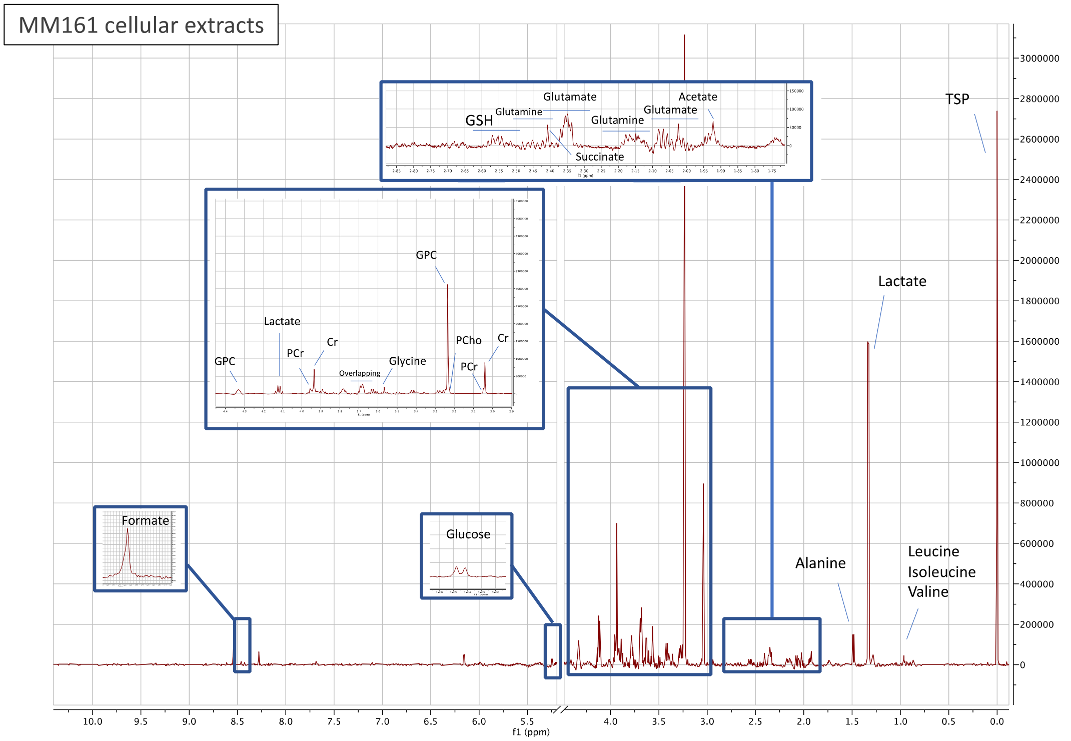

Figure S2. Typical cellular extracts ^{1}H-NMR spectra of the MM161 cell line.

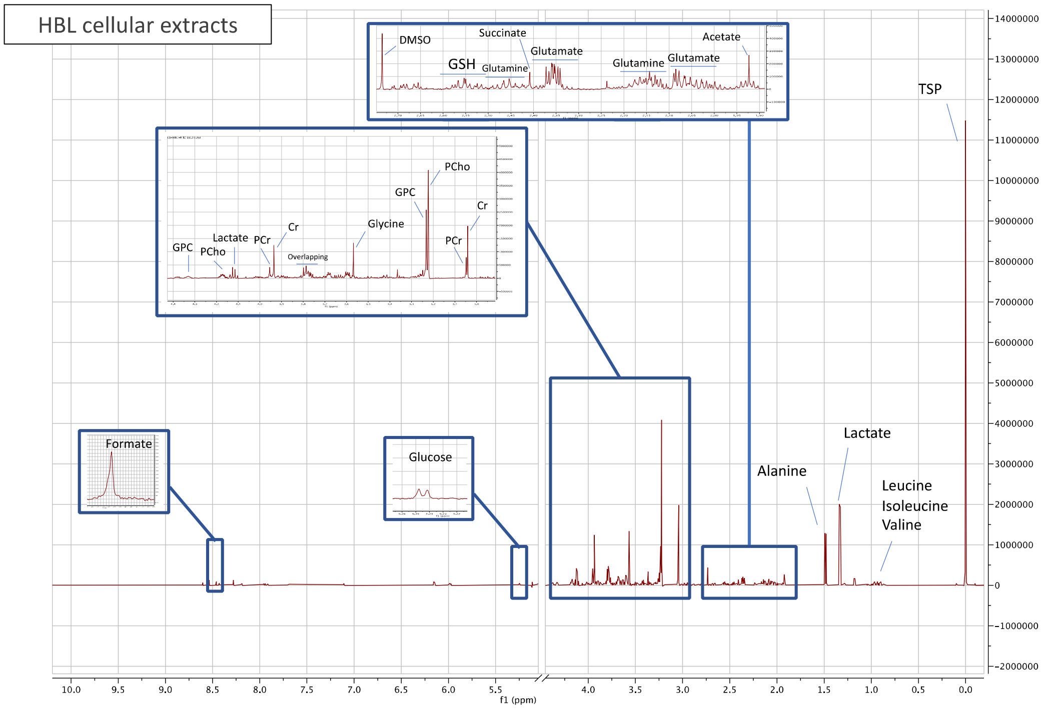

Figure S3. Typical cellular extracts ^{1}H-NMR spectra of the HBL cell line.

Table S1. Metabonomic signatures of the MM161 cells in various experimental conditions.

(A) Intracellular metabonomics signatures

AUC values	MM161 [(a)]	MM161 PIMA 72 h [(b)]	MM161-R PIMA [(c)]
**Lactate**	17.30 ± 3.36 * [(b)]	10.54 ± 1.52 * [(a)] *** [(c)]	25.96 ± 1.66 *** [(b)]
**Alanine**	1.19 ± 0.36	1.35 ± 0.20 * [(c)]	0.66 ± 0.13 * [(b)]
**Acetate**	0.79 ± 0.47	0.39 ± 0.18	0.59 ± 0.18
**Glutamine**	0.19 ± 0.14 * [(b)]	0.46 ± 0.14 * [(a)]	0.27 ± 0.11
**Glutamate**	1.82 ± 0.22	1.51 ± 0.30	1.52 ± 0.27
**GSH**	0.82 ± 0.23	0.72 ± 0.18 * [(c)]	1.30 ± 0.35 * [(b)]
**Succinate**	0.55 ± 0.11	0.40 ± 0.12 ** [(c)]	0.96 ± 0.12 ** [(b)]
**Aspartate**	0.12 ± 0.23	0.11 ± 0.10 * [(c)]	0.01 ± 0.01 * [(b)]
**Creatine**	5.86 ± 1.36 * [(b)]	7.68 ± 0.31 * [(a)] ** [(c)]	3.80 ± 0.53 ** [(b)]
**Phosphocreatine**	0.83 ± 0.25	1.22 ± 0.31	0.88 ± 0.09
**Phosphocholine**	0.72 ± 1.46 * [(c)]	1.55 ± 1.42	3.83 ± 1.96 * [(a)]
**Glycerophosphocholine (GPC)**	23.57 ± 4.15 * [(b)]	16.20 ± 3.04 * [(a)]	17.58 ± 2.31
**Glucose**	2.78 ± 0.51	2.40 ± 0.78	1.57 ± 0.90
**Glycine**	1.52 ± 0.31 * [(b)]	0.96 ± 0.44 * [(a)]	1.20 ± 0.30
**Formate**	0.09 ± 0.02	0.12 ± 0.04	0.13 ± 0.02
**Heatmap values**	**MM161 [(a)]**	**MM161 PIMA 72 h [(b)]**	**MM161-R PIMA [(c)]**
**Lactate**	0.67	0.41	1.0
**Alanine**	0.88	1.0	0.49
**Acetate**	1.0	0.49	0.75
**Glutamine**	0.41	1.0	0.59
**Glutamate**	1.0	0.83	0.83
**GSH**	0.63	0.55	1.0
**Succinate**	0.57	0.42	1.0
**Aspartate**	1.0	0.92	0.08
**Creatine**	0.76	1.0	0.49
**Phosphocreatine**	0.68	1.0	0.72
**Phosphocholine**	0.19	0.40	1.0
**Glycerophosphocholine (GPC)**	1.0	0.69	0.75
**Glucose**	1.0	0.86	0.56
**Glycine**	1.0	0,63	0,79
**Formate**	0.69	0.92	1.0

(B) Extracellular metabonomics signatures

AUC values	MM161 [(a)]	MM161 PIMA 72 h [(b)]	MM161-R PIMA [(c)]
**Glucose**	3.18 ± 0.06 * [(b)]	3.98 ± 0.45 * [(a)] * [(c)]	3.21 ± 0.27 * [(b)]
**Lactate**	91.30 ± 10.29 *** [(b)]	42.32 ± 13.35 *** [(a)]	67.31 ± 5.71
**Alanine**	1.47 ± 0.18	1.31 ± 0.20	1.25 ± 0.11
**Glutamine**	0.82 ± 0.29	0.63 ± 0.08 ** [(c)]	1.23 ± 0.09 ** [(b)]
**Heatmap values**	**MM161 [(a)]**	**MM161 PIMA 72 h [(b)]**	**MM161-R PIMA [(c)]**
**Glucose**	0.80	1.0	0.81
**Lactate**	1.0	0.46	0.74
**Alanine**	1.0	0.89	0.85
**Glutamine**	0.67	0.51	1.0

Area under the curve (AUC) of resonances from selected metabolites within intra- (**A**) or extracellular (**B**) ^{1}H-NMR spectra, presented as mean ± standard deviation. Heatmap values were calculated using univariate data conversion, dividing each metabolite AUC values by the highest one. Letters are used to tag groups (a: sensitive cells, b: sensitive cells exposed for 72 h to pimasertib, c: resistant cells exposed to pimasertib) and highlight the statistical significance resulting from the multiple Kruskal-Wallis test comparison of the groups as follows: * $p < 0.05$ ** $p < 0.01$ *** $p < 0.001$.

Table S2. Metabonomics signatures of the MM074 cells in various experimental conditions.

(A) Intracellular metabonomics signatures

AUC values	MM074 (a)	MM074 DABRA 24 h (b)	MM074 DABRA 72 h (c)	MM074-R DABRA (d)
**Lactate**	19.27 ± 2.25 * (b)	12.92 ± 3.10 * (a)	15.48 ± 1.14	17.34 ± 2.31
**Alanine**	4.13 ± 0.47 * (c)	2.09 ± 0.17 ** (d)	1.33 ± 0.28 * (a) *** (d)	7.40 ± 0.27 ** (b) *** (c)
**Acetate**	0.48 ± 0.64	0.91 ± 0.39	1.23 ± 0.69	1.18 ± 1.03
**Glutamine**	0.02 ± 0.04	0.28 ± 0.09 * (d)	0.34 ± 0.24 * (d)	0.01 ± 0.02 * (b) * (c)
**Glutamate**	2.85 ± 0.36 * (b) ** (c)	1.46 ± 0.17 * (a) * (d)	0.45 ± 0.24 ** (a) ** (d)	2.78 ± 0.36 * (b) ** (c)
**GSH**	0.75 ± 0.10	0.95 ± 0.19 ** (c)	0.22 ± 0.13 ** (b)	0.70 ± 0.17
**Succinate**	0.66 ± 0.08 * (b)	0.39 ± 0.10 * (a) *** (c)	1.03 ± 0.28 *** (b)	0.64 ± 0.07
**Aspartate**	0.02 ± 0.03 * (b)	0.10 ± 0.04 * (a) * (d)	0.31 ± 0.55	0.20 ± 0.53 * (b)
**Creatine**	4.17 ± 0.49 * (c)	4.94 ± 0.28 *** (c) * (d)	1.13 ± 0.24 * (a) *** (b)	3.40 ± 0.25 * (b)
**Phosphocreatine**	0.33 ± 0.15	0.62 ± 0.07 * (d)	0.36 ± 0.10	0.27 ± 0.28 * (b)
**Phosphocholine**	9.19 ± 1.52	7.63 ± 0.86 * (d)	2.47 ± 1.91 *** (d)	12.78 ± 0.91 * (b) *** (c)
**GPC**	10.49 ± 0.74 * (b) ** (c)	6.52 ± 0.83 * (a) * (d)	2.45 ± 1.04 ** (a) ** (d)	10.34 ± 0.86 * (b) ** (c)
**Glucose**	1.14 ± 0.45 * (c)	1.96 ± 0.51	4.17 ± 0.65 * (a) ** (d)	0.99 ± 0.58 ** (c)
**Glycine**	2.81 ± 0.48	1.60 ± 0.50 ** (d)	1.54 ± 0.25 ** (d)	5.68 ± 0.60 ** (b) ** (c)
**Formate**	0.19 ± 0.14 * (c)	0.14 ± 0.04 * (c)	0.30 ± 0.04 * (a) * (b)	0.15 ± 0.02
**Heatmap values**	**MM074** (a)	**MM074 DABRA 24 h** (b)	**MM074 DABRA 72 h** (c)	**MM074-R DABRA** (d)
**Lactate**	1.0	0.67	0.80	0.90
**Alanine**	0.56	0.28	0.18	1.0
**Acetate**	0.39	0.74	1.0	0.96
**Glutamine**	0.06	0.82	1.0	0.03
**Glutamate**	1.0	0.51	0.16	0.98
**GSH**	0.79	1.0	0.23	0.74
**Succinate**	0.64	0.38	1.0	0.62
**Aspartate**	0.06	0.32	1.0	0.65
**Creatine**	0.84	1.0	0.23	0.69
**Phosphocreatine**	0.53	1.0	0.58	0.44
**Phosphocholine**	0.72	0.6	0.19	1.0
**GPC**	1.0	0.62	0.23	0.99
**Glucose**	0.27	0.47	1.0	0.24
**Glycine**	0.49	0.28	0.27	1.0
**Formate**	0.63	0.47	1.0	0.50

(B) Extracellular metabonomics signatures

AUC values	MM074 (a)	MM074 DABRA 24 h (b)	MM074 DABRA 72 h (c)	MM074-R DABRA (d)
**Glucose**	3.81 ± 0.43	4.81 ± 0.21 * (d)	4.67 ± 0.22 * (d)	3.44 ± 0.31 * (b) * (c)
**Lactate**	68.38 ± 11.97 * (c)	36.27 ± 14.77	26.46 ± 12.96 * (a) ** (d)	69.16 ± 16.47 ** (c)
**Alanine**	2.41 ± 0.34 * (c)	1.46 ± 0.37	1.38 ± 0.19 * (a) * (d)	2.33 ± 0.50 * (c)
**Glutamine**	0.10 ± 0.09	0.39 ± 0.12	0.62 ± 0.12	0.24 ± 0.37
Heatmap values	**MM074** (a)	**MM074 DABRA 24 h** (b)	**MM074 DABRA 72 h** (c)	**MM074-R DABRA** (d)
**Glucose**	0.79	1.0	0.97	0.72
**Lactate**	0.99	0.52	0.38	1.0
**Alanine**	1.0	0.61	0.57	0.97
**Glutamine**	0.16	0.63	1.0	0.39

Area under the curve (AUC) of resonances from selected metabolites within intra- (**A**) or extracellular (**B**) ^{1}H-NMR spectra, presented as mean ± standard deviation. Heatmap values were calculated using univariate data conversion, dividing each metabolite AUC values by the highest one. Letters are used to tag groups (a: sensitive cells, b: sensitive cells exposed for 24h to dabrafenib, c: sensitive cells exposed for 72h to dabrafenib, d: resistant cells exposed to dabrafenib) and highlight the statistical significance resulting from the multiple Kruskal-Wallis test comparison of the groups as follows: * $p < 0.05$ ** $p < 0.01$ *** $p < 0.001$.

Table S3. Metabonomics signatures of the HBL cells in various experimental conditions.

(A) Intracellular metabonomics signatures

**AUC values**	**HBL (a)**	**HBL DASA 72 h (b)**	**HBL-R DASA (c)**
**Lactate**	9.00 ± 1.76 ** (b) * (c)	6.67 ± 0.49 ** (a)	6.92 ± 0.88 * (a)
**Alanine**	2.32 ± 0.19 *** (b)	4.02 ± 0.42 *** (a) * (c)	3.00 ± 0.18 * (b)
**Acetate**	0.52 ± 0.09 * (b)	0.32 ± 0.16 * (a)	0.45 ± 0.09
**Glutamine**	1.70 ± 0.12 ** (b) ** (c)	0.90 ± 0.15 ** (a)	0.84 ± 0.06 ** (a)
**Glutamate**	1.87 ± 0.19	1.86 ± 0.24	1.91 ± 0.16
**GSH**	1.11 ± 0.14	0.99 ± 0.26	1.05 ± 0.12
**Succinate**	0.24 ± 0.06	0.27 ± 0.05	0.23 ± 0.03
**Aspartate**	0.13 ± 0.02 * (c)	0.12 ± 0.02 * (c)	0.07 ± 0.02 * (a) * (b)
**Creatine**	3.72 ± 0.51	5.25 ± 1.74 * (c)	3.47 ± 0.48 * (b)
**Phosphocreatine**	1.57 ± 0.38 * (b)	2.63 ± 0.95 * (a)	1.76 ± 0.31
**Phosphocholine**	12.65 ± 1.24 * (b)	10.04 ± 1.65 * (a)	11.18 ± 1.12
**GPC**	5.97 ± 0.60 *** (b)	9.05 ± 1.04 *** (a)	7.09 ± 0.53
**Glucose**	1.83 ± 0.26	1.82 ± 0.36	1.86 ± 0.24
**Glycine**	0.09 ± 0.02 * (b) *** (c)	0.21 ± 0.05 * (a) * (c)	2.10 ± 0.22 *** (a) * (b)
**Formate**	0.12 ± 0.02	0.09 ± 0.02	0.11 ± 0.02
**Heatmap values**	**HBL (a)**	**HBL DASA 72 h (b)**	**HBL-R DASA (c)**
**Lactate**	1.0	0.74	0.77
**Alanine**	0.58	1.0	0.75
**Acetate**	1.0	0.62	0.87
**Glutamine**	1.0	0.53	0.49
**Glutamate**	0.98	0.97	1.0
**GSH**	1.0	0.89	0.95
**Succinate**	0.88	1.0	0.85
**Aspartate**	1.0	0.92	0.54
**Creatine**	0.71	1.0	0.66
**Phosphocreatine**	0.6	1.0	0.67
**Phosphocholine**	1.0	0.79	0.88
**GPC**	0.66	1.0	0.78
**Glucose**	0.98	0.98	1.0
**Glycine**	0.04	0.10	1.0
**Formate**	1.0	0.75	0.92

(B) Extracellular metabonomics signatures

**AUC values**	**HBL (a)**	**HBL DASA 72 h (b)**	**HBL-R DASA (c)**
**Glucose**	4.67 ± 0.11 *** (c)	4.44 ± 0.20 * (c)	3.90 ± 0.23 *** (a) * (b)
**Lactate**	50.82 ± 6.56 *** (b)	33.88 ± 2.93 *** (a)	41.54 ± 3.23
**Alanine**	1.95 ± 0.23	1.82 ± 0.18	1.84 ± 0.24
**Glutamine**	1.15 ± 0.16 * (b) *** (c)	0.56 ± 0.07 * (a)	0.43 ± 0.06 *** (a)
Heatmap values	**HBL (a)**	**HBL DASA 72 h (b)**	**HBL-R DASA (c)**
**Glucose**	1.0	0.95	0.84
**Lactate**	1.0	0.67	0.82
**Alanine**	1.0	0.93	0.94
**Glutamine**	1.0	0.49	0.37

Area under the curve (AUC) of resonances from selected metabolites within intra- (**A**) or extracellular (**B**) ^{1}H-NMR spectra, presented as mean ± standard deviation. Heatmap values were calculated using univariate data conversion, dividing each metabolite AUC values by the highest one. Letters are used to tag groups (a: sensitive cells, b: sensitive cells exposed for 72h to dasatinib, c: resistant cells exposed to dasatinib) and highlight the statistical significance resulting from the multiple Kruskal-Wallis test comparison of the groups as follows: * $p < 0.05$ - ** $p < 0.01$ - *** $p < 0.001$.

Table S4. Median centered Log2 data of the relative protein levels between the HBL, MM074 and MM161 resistant and sensitive counterparts.

Protein name	Sample description					
	HBL	HBL-R	MM074	MM074-R	MM161	MM161-R
DUSP4	−1,903073954	−0.535891047	−0.281792955	0.955274349	0.658281949	0.281792955
HIF1A	−0.574084212	−0.094590894	0.232850745	1.506291346	0.094590894	−0.305689909
MAPK1,3	−1.029546585	−0.99650041	−0.126599225	1.057120703	0.126599225	0.947461899
PLCG1	0.152585769	0.097659941	−1.476234731	−0.137862469	−0.097659941	0.702727645
Tau	−0.93692358	−0.053104712	−1.052763994	0.053104712	0.267849423	0.278592465
ATRX	−0.25039738	−0.006105834	0.452982029	−0.611029244	0.006105834	0.705620881
Caveolin 1	0.157388396	0.269891226	1.647437576	−0.157388396	−0.169231058	−0.199839293
EPHA2	0.093669683	−0.560703649	2.627886222	0.38645617	−0.212129776	−0.093669683
EPHA2	−0.190491324	−0.302815025	1.787832647	0.375551256	−0.144898341	0.144898341
Rho GTPase activating protein 45	−1.843519301	−1.2423631	0.768232202	−0.768232202	1.38918177	2.171147282
PARG	−0.404217551	−0.080766764	1.242504695	−0.228860887	0.080766764	0.126884143
MLKL	−0.104545873	−0.193535146	0.104545873	−1.171105769	1.91805893	1.93223713
STAT5A	−0.371694476	−0.241862147	0.241862147	−0.797745855	1.181642243	0.796311949
BCL2L11	−0.629146848	0.426960563	−0.744560383	−0.131129775	0.541157293	0.131129775
DNMT1	−0.242796493	0.790347808	−0.721359808	−0.405990615	0.242796493	0.311450513
FOXM1	−0.726127382	0.408681713	-0.726989284	−0.408681713	0.685997615	0.81260914
MSH6	−0.528605186	0.756253597	−0.752770362	−0.321893606	0.321893606	0.657301197
RB1	−0.609487278	0.749124156	−1.009323131	−0.675610405	0.614782846	0.609487278
AKT 1/2/3	2.476047665	0.280506987	0.090616579	−0.291949794	−0.090616579	−0.178265815
AKT 1/2/3	1.213853733	−0.001435672	−0.084215398	0.009653353	−0.185219727	0.001435672
GYS1	0.820206376	−0.228717023	0.25539653	0.228717023	−0.329946576	−0.447764884
p21	1.374127741	−0.91393387	1.343531022	0.91393387	−2.520938357	−2.644286415
RPS6KA1	−0.112089914	−1.912268939	0.564518527	0.175202995	−1.087055761	0.112089914
SLC1A5	0.800748501	−0.428186856	0.062005418	−0.062005418	0.2772064	−0.362238978
SRC	0.362008929	−3.050474284	0.189322812	−0.227050453	0.655644903	−0.189322812
Rps6	−0.112089914	−1.912268939	0.564518527	0.175202995	−1.087055761	0.112089914
Rps6	0.504791002	−0.9750599	0.623395095	0.245045029	−1.781288812	−0.245045029
ZAP70	2.4889699	−0.00801409	0.120294259	−0.060367097	0.00801409	−0.016139345
CDC6	−0.104391808	0.138162769	−0.72861176	−0.110228026	0.104391808	1.109268053
ER-a	−0.35330408	−0.016668567	−0.267597011	0.016668567	0.393382995	2.962696234
Histone H3	−0.104545932	0.152519008	−0.097293528	0.097293528	−0.139922002	2.169434008
MAP2K1,2	−0.201238483	−0.285805012	0.475508867	0.013175291	−0.013175291	1.001260984
HK2	−0.005088175	0.005088175	−0.419560549	0.502940827	0.80444892	−0.518766725
c-kit	1.791504654	2.26639649	−0.535834229	−0.478565734	0.478565734	−2.789795411
COX1	0.640978672	0.503342035	−0.550204093	−0.503342035	0.856353098	−0.71268049
